# Inside the “Black Box” of a Knowledge Translation Program in Applied Health Research

**DOI:** 10.1177/1049732315580104

**Published:** 2015-11

**Authors:** Janet Heaton, Jo Day, Nicky Britten

**Affiliations:** 1 University of Exeter, Exeter, United Kingdom

**Keywords:** community-based programs, complexity, knowledge transfer, knowledge utilization, program evaluation, qualitative analysis, research, collaborative, research, dissemination and utilization, research, qualitative

## Abstract

In this article, we present the findings of a participatory realistic evaluation of a 5-year program of health care research intended to promote the translation of knowledge into routine clinical practice. The program was one of the nine pilot Collaborations for Leadership in Applied Health Research and Care funded by the English National Institute for Health Research between 2008 and 2013. Our aim was to delineate the mechanisms by which, and circumstances in which, some projects carried out under the program achieved success in knowledge translation while others were frustrated. Using qualitative methods, we examined how closer collaboration between academics and clinicians worked in four purposefully chosen case studies. In a synthesis of the findings, we produced a “black box” model of how knowledge translation was enabled by the activation of nine mechanisms. These are summarized in the form of five simple rules for promoting knowledge translation through collaborations based on principles of coproduction.

“Knowledge translation” and “knowledge utilization” are just two of many terms that have been used to describe the process by which knowledge from research is implemented into practice. In applied health research (AHR), it is generally recognized that more could be done to speed up and spread the application of evidence from studies into routine clinical practice, thereby improving patient outcomes. However, there is no consensus over how to close this gap in knowledge and practice. In North America, Europe, and Australia, agencies that fund AHR have approached this problem in different ways (Tetroe et al., 2008).

In England, like many other developed countries, health care research has often been led by academic researchers with minimal involvement of those who commission, provide, and use health services. In 2008, the National Institute for Health Research (NIHR) funded a national 5-year pilot program of AHR that was intended to bridge this cultural divide. As the name suggests, the NIHR “Collaborations for Leadership in Applied Health Research and Care” or “CLAHRCs” were founded on a partnership approach to knowledge translation. They were established locally, to enable researchers in universities and those who work in and use the National Health Service (NHS), to jointly undertake and implement research.

The CLAHRCs’ approach is similar to the “interactive” model of knowledge utilization described by Weiss (1979, p. 428). In this model, social scientists, decision makers, and subjects of policy (among others) all work together and pool their intelligence to respond to social issues. This way of working has been promoted by funders of health care research in Canada for many years. Several evaluations have found closer working between researchers and end users of knowledge to be a facilitator of the diffusion of innovation in health services (Conklin, Hallsworth, Hatziandreu, & Grant, 2008; Elliot & Popay, 2000; Innvaer, Vist, Trommald, & Oxman, 2002). However, a few studies have been inconclusive (Kothari, Birch, & Charles, 2005; Kothari & Wathen, 2013) and others have argued that there is a need to better understand the variable nature of partnership models of AHR before any particular model is privileged (Mitchell, Pirkis, Hall, & Haas, 2009).

In this article, we present the findings from an internal evaluation of one of the nine pilot CLAHRCs, in which we examined the nature and workings of the “partnership” or “interactive” approach it adopted. Below, we describe in more detail the background, aims, and configuration of the “NIHR Collaboration for Leadership in Health Research and Care for the South West Peninsula,” otherwise known as “PenCLAHRC.”

## Overview of the Program

NIHR commissioned the CLAHRCs following the publication of high-level reports that highlighted two important gaps in the translation of evidence from research into routine clinical practice in the NHS (Department of Health, 2007; Treasury, 2006). The first gap was in the conversion of ideas from basic research into the development of new products and therapies; the second was in the uptake of tried and tested interventions in the NHS. The brief for the CLAHRCs was to close the second translational gap by conducting and implementing high-quality research, and by increasing the capacity of NHS organizations to utilize evidence.

Initially, nine pilot CLAHRCs were awarded funding from 2008 for up to 5 years at a cost of £90 million to NIHR, matched in kind by the partner universities and NHS organizations (Walshe & Davies, 2013). In 2014, the CLAHRC initiative was continued following early positive findings of evaluations of the pilot programs nationally (Currie, Lockett, & Enany, 2013; D’Andreta, Scarbrough, & Evans, 2013; Rycroft-Malone et al., 2013; Soper et al., 2013). This time, NIHR funded 13 CLAHRCs over a further 5 years, incorporating some new and some geographically reconfigured collaborations (including PenCLAHRC), at a total cost of £124 million. These were commissioned in areas where Academic Health Science Networks (AHSNs) had also been set up in a related initiative to accelerate the adoption and spread of innovation in the NHS (Nicholson, 2011).

In the initial pilot phase, the CLAHRCs were characterized as local experiments in knowledge translation. Each program was encouraged by NIHR to innovate and adapt to fit the needs of their population. The NIHR was also clear that it expected it would take more than 5 years for real culture change to be achieved. However, in line with its brief, it did expect the pilot programs to be able to deliver research outcomes in the form of academic publications, research income and increased research capacity, as well as implementation impacts in the form of improvements in local health services and patient outcomes.

### The PenCLAHRC Pilot

PenCLAHRC was one of the nine original pilot programs. It was launched in October 2008 following an award of £10 million from NIHR and the equivalent in matched funding from its partners. Two higher education institutions and 13 NHS trusts (health service providers) in the far southwest peninsula of England were involved in the collaboration. The former included the Universities of Exeter and Plymouth, and their then jointly run Peninsula College of Medicine and Dentistry. The latter included NHS South-West, which was the Strategic Health Authority overseeing trusts in the area; all the acute, mental health, and primary care trusts in the region; and the ambulance trust for the southwest.

Most of the CLAHRCs proposed to carry out designated research and implementation projects on specific knowledge translation topics. However, in PenCLAHRC, a different approach was taken. Here the funding was used to institute and test a system for the design, conduct, and implementation of AHR on a collaborative basis. The system was built around the notion of “Engagement by Design©.” This meant end users of research—including clinicians, managers, commissioners, patients, and the public—working in close collaboration with researchers in academic institutions to conduct research on topics that were directly important to these end users.

End users were actively involved in proposing research questions for PenCLAHRC to adopt and turn into applications for external research funding, or into business cases for evidence-based implementation work with local NHS trusts. There were different routes by which projects could be suggested. One route was through a formal “question-generation and prioritization” process. Five rounds of this process were held, where people were invited to submit questions on topics that particularly mattered to local end users. A panel of stakeholders reviewed the questions using agreed criteria and prioritized them for adoption by PenCLAHRC. People could also suggest questions at other times, enabling urgent or opportunistic ideas to be given prompt consideration in-between rounds of the more structured process.

As well as pump-priming projects, PenCLAHRC also funded activities and groups to facilitate end users’ participation in the program and to increase research capacity in the region. For example, “question-generation workshops” and “evidence-based practice” courses were run for clinicians, patients, and the public. These were designed to help participants to develop research questions, and to improve their research knowledge and skills. A patient and public involvement group, which called itself “PenPIG” (“Peninsula Patient Involvement Group”), was also set up to promote their engagement in the program.

Finally, several new staff were appointed to PenCLAHRC posts. These included six clinicians from acute and primary care trusts who were seconded from their NHS roles for 2 days a week to act as “Locality Leads.” Their role was to foster relationships between their local trusts and the wider collaboration, as well as to help solicit questions from their NHS colleagues. New methodologists were also appointed, with expertise in evidence synthesis, modeling, and quantitative and qualitative methods. So too were various support staff, including a manager for operations and finance, a patient and public involvement facilitator, and project facilitators.

## Evaluation Aims and Conceptual Framework

In its original bid to NIHR, PenCLAHRC included provision for a formative internal evaluation of the pilot program, as did several other CLAHRCs (Martin et al., 2011). The overall aim of the internal evaluation was to examine whether the program succeeded in producing, implementing, and/or improving capacity for AHR in southwest England, and to explain why it succeeded or not.

We used “realist” or “realistic evaluation” for this purpose (Pawson & Tilley, 1997/2008). Founded on realist epistemology, it was developed to enable evaluators to build theories about whether, where, and why programs do what they are “supposed to do” (STD), based on empirical observations (Tilley, 2004, p. 257). It has been widely used to evaluate health care programs (e.g., Byng, Norman, & Redfern, 2005; Greenhalgh et al., 2009). In our “participatory” version, we actively involved the senior managers of PenCLAHRC in the design of the evaluation. Through our participation in the pilot program, we also helped to shape PenCLAHRC as it developed.

In realistic evaluation, it is assumed that programs are “theories incarnate” (Westhorp et al., 2011, p. 1). That is, they are based on ideas, conjectures, and suppositions about how a program will bring about a change, even though the theories behind it might not always be explicit. The challenge for the realist evaluator is to identify, make manifest, test, and refine theories on what it is about a given program that might bring about the desired change.

Evaluators may develop theories from various sources. These include program architects’ and participants’ own “folk theories” or “folk conjectures” about how a program is meant to work (Pawson & Manzano-Santaella, 2012, p. 181); formal social science theory, results of previous evaluations, and common sense may also be drawn upon (Tilley, 2000). In an iterative process, the evaluator progressively elaborates and empirically tests these working theories, using appropriate methods and available knowledge, to further develop and improve understanding of whether and why a program works in practice.

Various strategies for surfacing, testing, and refining program theories have been documented elsewhere (Pawson & Manzano-Santaella, 2012; Pawson & Tilley, 1997/2008). Essentially, the protocol involves expressing candidate theories in the form of context–mechanism–outcome (CMO) propositions. These delineate the outcomes that are hypothesized to result from the action of mechanisms triggered by a program in given contexts. Mechanisms are defined as “agents of change” (Pawson, 2013, p. 115). They are the means by which programs are thought to influence the “reasoning” and, ultimately, the behavior of program subjects (Pawson, 2013, p. 115).

Candidate theories are tested by examining if a program works as envisaged in various contexts. Data are purposefully gathered in case studies to determine what it is about the program (M) that works for whom (O) in what circumstances (C). These empirical observations are then used to refine the candidate theories accounting for how a program works. Ideally, mid-range theories are developed that capture underlying regularities in CMOs in social life.

We broadly followed this protocol in our evaluation of PenCLAHRC. However, we adapted part of it in the following respect. In the process of theory-building, we found it difficult to directly link the outcomes we observed, some of which were quite broad or partial, to the interaction of particular mechanisms and contexts. We also became concerned that breaking down and expressing the CMOs in the usual form of CMO1, CMO2 propositions, and so on might inadvertently give the impression that they were linear causal chains or pathways. Instead, we explored other ways of conceptualizing and mapping the relationships between CMOs that better captured the complexity of these interactions.

For this purpose, we drew on the work of Plsek (2001) and other complexity theorists (Byrne, 2013; Geyer & Rihani, 2010) to help model the relations between CMOs. Complexity theory is concerned with the study of complex adaptive systems in the natural and social world, such as ecosystems and health care systems. Unlike orderly systems that operate in machine-like ways, and chaotic systems that have no discernible order, complex systems are driven by simple rules that generate complex forms of behavior. The interaction of agents in complex systems leads to outcomes that have emergent and nonlinear qualities, whereby small actions can have big effects and vice versa. Complex systems also have the capacity to self-organize and adapt in response to changes in their local environments. In these ways, they shape and are shaped by their environment in a recursive process.

Drawing on these ideas, we conceptualized PenCLAHRC to be a collaboration of complex adaptive systems (health care organizations and academic institutions) and its projects to be similar nested systems. We attempted to model the operation of mechanisms in contexts, and the generation of outcomes, in a way that represented the emergent and nonlinear qualities of these complex interactions. This meant that we did not try to attribute or apportion the outcomes of the program to particular mechanisms and contexts. Instead, we merely sought to open the “black box” of PenCLAHRC and, through observation of the program in action in a range of circumstances, to delineate those mechanisms that, where active, appeared to make a difference to the success of the program. We saw this approach as compatible with realist evaluation, providing a way of dealing with the “problem of complexity” that the methodology is critically concerned with (Pawson, 2013, p. xv).

## Method

The evaluation was carried out over the 5 years of the pilot program. We used mainly qualitative methods in three phases: first, to surface and articulate the various STD theories that underpinned the program; second, to elaborate and test one set of key theories about closer collaboration in four case studies; and, third, to synthesize the findings from our various observations and describe the simple rules that characterized the ways in which closer collaboration was enacted in the relatively successful projects undertaken in PenCLAHRC.

As we describe in more detail below, we drew on various sources of data from the program in these phases of theory-building, including interviews with program stakeholders and project participants, and official documents. In the last phase, we also drew on existing literature on the concept of “coproduction” (Ostrom, 1996) to interpret the results and develop a potential mid-range theory of why this style of closer collaboration helped to promote knowledge translation in PenCLAHRC.

### Phase 1: Surfacing of Program Theories

We began our theory-building by eliciting the program stakeholders’ views on the goals of PenCLAHRC, the strategies being used to achieve them and what its success might depend upon. A total of 77 semistructured interviews were carried out over the first 2 years of the pilot program with 54 stakeholders from the NHS, academia and PenPIG. These included 9 interviews with 5 senior figures who were involved in compiling the original bid and in managing the program. We also read the original proposal to NIHR, annual reports and other official documents, to identify any statements that provided insights into how the program was hypothesized to work.

All the interviews were audio-recorded, with permission, transcribed verbatim and anonymized. After reading the transcripts, we developed a set of codes for indexing stakeholders’ references to potential CMOs associated with the program. For example, some of them highlighted the different priorities and timescales of researchers and clinicians in their respective academic and NHS organizations (C), and how this might inhibit successful collaboration. They also considered whether strategies such as the question-generation and prioritization process were likely to trigger change and enable clinicians to be more active in driving research (M).

These and other relevant themes were coded on a computer using specialist software. In our analysis of these data, we found that the 5 program architects had the most comprehensive and detailed knowledge of the design and operation of PenCLAHRC as a whole, compared to the other stakeholders who had a more partial understanding. We therefore focused our analysis on the interviews with the former, and the official PenCLAHRC documents, to identify their theories about the program. Through detailed thematic analysis of these data, we compiled a list of 21 STD hypotheses. At this stage, we expressed the hypotheses in a way that was similar to how they had been originally stated by the architects. The interviews with the other stakeholders were used as a potential source of information for refining or building alternative theories, and for identifying any concerns over how the program was progressing.

We presented the list to a group of senior managers in PenCLAHRC, which included some of the original program architects. They were invited to comment on the veracity and completeness of the hypotheses, and to nominate ones that they would most like the internal evaluation team to research in more depth. This resulted in a short list that we divided into four sets of theories pertaining to “closer collaboration,” “infrastructure,” “patient and public involvement,” and “implementation.” Because we did not have the resources to look at all four sets, and given other embedded evaluations were planned in two of these areas, we decided to focus on the theories of “closer collaboration” that were so central to the program.

### Phase 2: Elaboration of Selected Program Theories

In the next phase, 4 PenCLAHRC projects were selected as case studies for testing the theories of closer collaboration that we had identified. The same group of managers as before nominated the projects. They were given a closer collaboration proposition we had compiled from the Phase 1 work. This stated that, compared with the traditional ways in which AHR had been undertaken, “research driven by end users (both professionals and patients/public), carried out in partnership with methodologists, is more likely to produce evidence that meets end users’ specific information needs.” We asked the managers to nominate 2 projects that they thought at the time provided some support for the hypothesis, and 2 that as yet did not, from around 50 that were established or completed at the time.

We then asked the leads of the nominated projects whether they and their project teams would be willing to take part in the evaluation, which they were. The projects were in 4 different clinical areas: thrombolysis following acute ischemic stroke; pelvic floor muscle training (PFMT) for women with urinary incontinence; administration of tranexamic acid (TXA) in trauma; and falls prevention for the frail elderly. They involved various local collaborations between researchers from two universities, hospital clinicians from different specialisms, community-based clinicians, and paramedics. Further information on the origins, aims, constitution, and progression of the projects is provided in Table 1.

**Table 1.** The Four Case Study Projects.

Stroke Thrombolysis	TXA in Trauma
Origin: A question submitted by a clinician in the first round of the question-generation and prioritization process in 2009 Aim: To minimize the time between the onset and treatment of acute ischemic stroke. The project was split into two parts: (a) a study of the effects of extending the license for administration of treatment from 3 to 4.5 hr; (b) computer simulation modeling of the stroke pathway at one hospital to identify scope for improvements Partners: A university, acute stroke unit, emergency department, ambulance trust, and regional stroke network Progress: A prealert system was introduced in a local hospital; 4 times more patients were treated in half the time postimplementation; two journal articles were published; led to spin-off projects in other local centers	Origin: In 2011, the PenCLAHRC director met the TXA trial lead by chance and they formed the idea for the project Aim: To implement the use of TXA for trauma patients (where TXA is administered twice, once by paramedics and once in the emergency department) in southwest England Partners: A university, ambulance trust, and 11 emergency departments in southwest England Progress: By late-2011, TXA in trauma had been implemented across southwest England, followed by 9 of the 11 ambulance trusts nationwide; in 2012, the ambulance trust won a national innovation award for the work; over 70 patients received TXA over 13 months following implementation, with numbers gradually increasing over time; no academic publications to date (but were planned)
PFMT	Falls Prevention
Origin: A question submitted by a clinician in the second round of the question-generation and prioritization process in 2010 Aim: Initially, it was to implement and evaluate a package of PFMT delivered in primary care to treat urinary incontinence. It required funding from commissioners of primary care to implement a training package in general practices. The business case was approved in autumn 2011 for an expanded project, including prevention as well as treatment Partners: A secondary care NHS organization, primary care trust, commissioning service, and university Progress: Stalled in 2012 when NHS reforms meant partners had to regain funding agreement; the evaluation protocol was published	Origin: Identified by senior managers in PenCLAHRC as a potential project and included in the original bid to NIHR in 2008 Aim: Initially, it was to conduct a trial of a multifactorial falls prevention program in primary care. This was negated by publication of a Cochrane Review and trial research from the USA. The focus shifted to implementation of evidence and to frailty in the elderly Partners: A university, NHS trust, and representatives from primary and secondary care Progress: Two systematic reviews published; led to a service review of fall prevention activities in the southwest and to the reestablishment of regional falls network and review of falls exercise groups; unsuccessful bids for external research funding

*Note.* TXA = tranexamic acid; PFMT = pelvic floor muscle training; NIHR = National Institute for Health Research; NHS = National Health Service.

Members of the project teams were interviewed using a semistructured topic guide to obtain their views on what the projects were supposed to do, how the collaborations had worked, and what they had achieved in each case. A total of 28 interviews were carried out with researchers, clinicians, project facilitators, and others involved in the projects. All the interviews were audio-recorded, with permission, and processed as before. We also collected and read documents about the projects for background information and for monitoring the progress of the work. These included notes of meetings, related research proposals, published articles, press releases, and information disseminated via the PenCLAHRC website.

These data were read and indexed on a computer using 12 codes that were developed to capture the participants’ reasoning around whether and why the projects had done what they were STD. The index included codes for “who STD,” “did it do STD,” “unintended outcomes,” “why did it do STD,” “why didn’t it do STD,” and “what ifs.” In an ongoing process of data reduction and display, we then manually summarized the contexts, mechanisms, and outcomes linked to the theories. All the authors were involved in the process of coding and data extraction, and/or cross-checking the results.

In our analysis of these data, we used a form of force-field analysis (Baulcomb, 2003) to visually map the forces for and against the activation of mechanisms in each of the projects. These included positive and negative forces emanating from the local and wider contexts of the projects, such as the significant national reorganization of primary care in England that occurred during the program, which stymied the progress of some of the projects.

As the evaluation progressed, we also continued to monitor the outcomes achieved by the projects over time. These included research publications, external funding, changes to services and any benefits to patients reported to date. By the end of the evaluation, 3 of the projects had been officially completed; only the PFMT project was still ongoing, after considerable delays. Two of the completed projects (Stroke and TXA) also had embedded evaluations that were not complete and hence were still revealing outcomes beyond the term of the present evaluation. We therefore recognized that the outcomes data available to us at the time of writing would be partial and incomplete.

### Phase 3: Synthesis of Results

In the final phase of the evaluation, we compared the findings across the 4 case studies, looking for what distinguished the more successful projects from the relatively less successful ones. We identified 9 mechanisms of closer collaboration that were active in two or more of the case studies. For each mechanism, we completed a grid summarizing the nature of the mechanism and the contextual factors or forces that helped or hindered it, by each project. We also reviewed the outcomes achieved to date by each project against their stated aims and the overall goals of the program. Again all authors jointly carried out this process of data reduction and display, checking and agreeing the final versions of the charts.

Through this process, we produced a synthesis and associated model of the key mechanisms by which, and circumstances in which, some of the projects achieved knowledge translation. In keeping with our complexity-informed approach to realist evaluation, we summarized the action of these mechanisms in the form of 5 simple rules. At this stage, we noticed some similarities between the mechanisms of closer collaboration that we had identified and the concept of “coproduction” (Ostrom, 1996). In the final step of our theory-building, we considered the relevance of the theory of coproduction as a possible mid-range theory for interpreting our findings and explaining why knowledge translation was more readily accomplished in some PenCLAHRC projects than in others.

### Ethics

In England, ethical approval was required for research studies but not evaluations or audit studies at the time of this work. We consulted the Chair of a NHS Local Research Ethics Committee who confirmed that approval was not required in this case. Participants were given information about the evaluation before the interviews and consented to the interviews being recorded and their views being reported anonymously.

## Results

We found considerable variation in the progress that the projects had made. The Stroke and TXA projects had achieved more success in meeting end users’ information needs than the PFMT and Falls projects (see Table 1). In the former projects, evidence had been successfully used to change and improve the ways in which local services were delivered, leading to more patients receiving the associated evidence-based treatment, and in a shorter time. The implementation of TXA in trauma had also been rapidly reproduced nationally in England. Not only were the professional end users pleased with the outcomes of the Stroke and TXA projects, but they had also gone on to be involved as partners in further projects in the collaboration.

**Figure 1.**
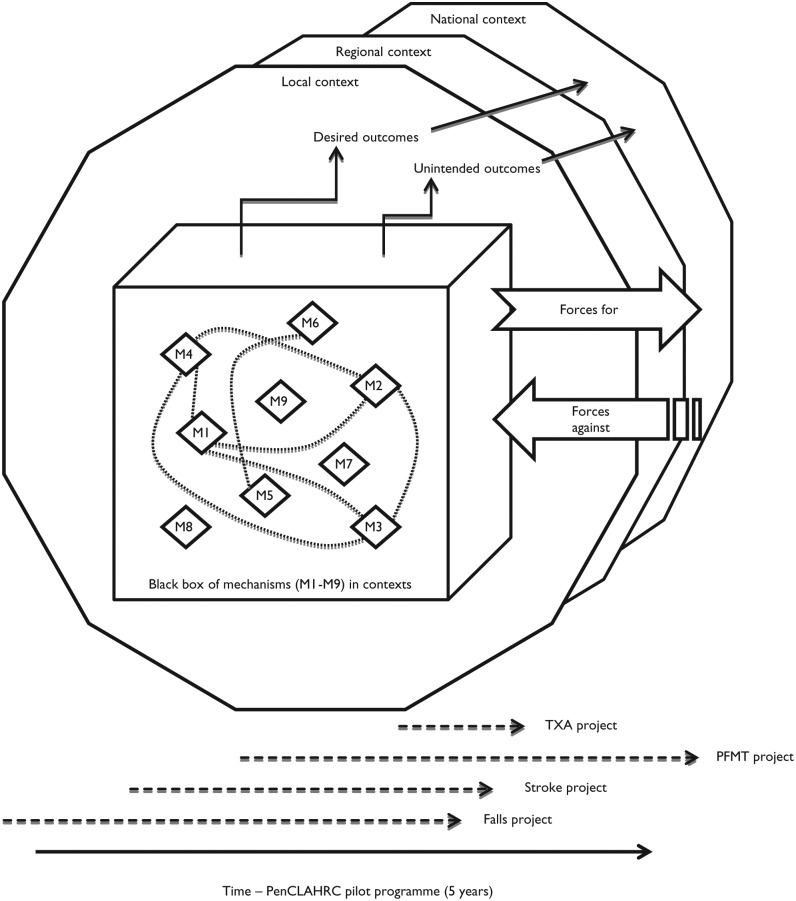
The “black box” model of mechanisms of closer collaboration in PenCLAHRC. *Note.* TXA = tranexamic acid; PFMT = pelvic floor muscle training.

In our synthesis of the mechanisms at play in the case studies, we discerned 9 mechanisms of successful closer collaboration, and various contextual factors that had helped or suppressed their activation by the program. In line with our complexity-informed approach to realist evaluation, we developed a “black box” model of the relations between these CMOs. The model is shown in schematic form in Figure 1.

At the heart of the model is a black box containing the 9 mechanisms (M1–M9) we observed. Emerging from the box are the desired outcomes achieved by the projects, as well as some unintended outcomes. These outcomes were conceptualized to emerge from the interaction of one or more of the mechanisms and the contexts (local, regional, national) in which the projects and program operated. Although these interactions were complex and indeterminate in nature, they were shaped by simple rules that were discernible. Thus, in our thesis, when a number of the mechanisms were active, end users’ information needs were more likely to be met; when they were not, this outcome was less likely to be achieved. Whether the mechanisms were active or not depended on how people acted in the contexts of the individual projects, where different factors helped and/or hindered their efforts.

Below we describe the five simple rules in detail, giving examples of whether the associated mechanisms were activated in the contexts of the projects. For convenience, the simple rules and associated mechanisms are summarized in Table 2.

**Table 2.** Five Simple Rules of Closer Collaboration and Associated Mechanisms (Ms).

Rule 1: Base AHR on Coproduction Through Closer Collaboration	
M1. Local end user driven	Local end users are placed at the heart of AHR. They are involved in driving research, so that it focuses on real-life issues that are relevant and important to them, and throughout the research life cycle
M2. Meeting of minds	End users and researchers find a common and coherent objective around which they coalesce. Their commitment and enthusiasm is matched with strategic support from their respective organizations
M3. Knowledge appetite	End users and researchers are open and receptive to melding different forms of knowledge. This includes clinicians’ knowledge of routine clinical practice, patients’ experiential knowledge, and researchers’ methodological expertise. Each recognizes and values what the other partners can contribute
M4. Game changers	End users and researchers find new and more productive ways of doing and implementing research through working in collaboration. They see wider potential for the new way of working
Rule 2: Establish Small Strategic Teams Led by Strong Facilitative Leaders	
M5. Facilitative leadership	Project teams are led by one or more leaders, who are regarded within and outside the team as credible and having real clout, connections, drive, enthusiasm, and tenacity. A facilitative style of leadership works well to involve partners, and to coproduce and mobilize knowledge for implementation
M6. Small strategic core	Project teams are formed around a small strategic core of end users and researchers from the partner organizations involved in the project
Rule 3: Harness and Develop Respective Assets	
M7. Creative assets	Partners harness existing and build up new assets to facilitate the conduct and implementation of AHR. “Assets” include: people with particular knowledge and skills; continuing professional development opportunities; routine data; websites for sharing learning; publications
Rule 4: Promote Relational Adaptive Capacity	
M8. Relational adaptive capacity	Learning from local AHR is actively shared with and adapted to kindred settings or populations in other areas (locally, nationally, internationally)
Rule 5: Remember—The End User Is King!	
M9. End user is king!	Partners recognize that the key change agents are not the program “makers and shakers” and the strategies they introduce but rather the agents on the ground and how they respond to the opportunities afforded by the program to change how AHR is routinely carried out and implemented

*Note.* AHR = Applied Health Research.

### Rule 1: Base AHR on Coproduction Through Closer Collaboration

From the beginning, the architects of PenCLAHRC had a strong vision about the nature of the second translational gap and how best to close it. The root problem was conceived to be the disconnection between the worlds of the researchers and the end users of research, with the former producing evidence that the latter were either not aware or interested in. PenCLAHRC was intended to engender a systemic change in the ways in which researchers carried out AHR. Through “Engagement by Design©,” the architects of PenCLAHRC sought to enable researchers and end users to work together at all stages in the design, conduct, and implementation of research. By engaging end users in the coproduction of research, it was thought that they would be more likely to use the evidence to aid their decision making as commissioners, managers, clinicians, or patients.

In the case studies, we observed that while they all involved closer collaboration between researchers and clinicians, the more successful projects were characterized by a more active style of engagement. This was where the partners were enabled by the program to jointly agree and pursue a research topic to their mutual advantage. There were 4 mechanisms that distinguished this way of working, which we describe below. These mechanisms form the basis of our first simple rule: that AHR should be based on coproduction through closer collaboration.

One of the associated mechanisms was whether the projects were “local end user driven” (shown in Figure 1 as “M1”). Members of 3 of the case studies believed that local end users were driving their projects or playing a crucial part in leading on some aspect of them. Both the Stroke and PFMT projects were based on questions that had been developed by local clinicians and taken on by PenCLAHRC through the question-generation and prioritization process. The original question that formed the basis of the PFMT project had also been expanded following the intervention of the local primary care trust, to include prevention as a well as treatment of urinary incontinence. Although the TXA project did not originate from a local end user, members of the team thought that the local ambulance trust and clinicians from two local emergency departments were leading in implementing the results across the region.

The exception was the Falls project. This was one of the 4 projects adopted by PenCLAHRC at the outset of the program to try and achieve some “quick wins” by capitalizing on existing ideas for research and implementation projects that fitted the aspirations of the program. It was inspired by the work of a local rural general practice that had achieved a much lower rate of falls among the elderly compared to other practices in the area. Senior managers in PenCLAHRC proposed to develop a similar intervention and try it with a receptive local trust with a view to rolling it out more widely in the southwest. The proposal was included in the original bid to NIHR as the sole exemplar of how PenCLAHRC might evaluate local innovative practices and spread them more widely with potentially many benefits for patients and cost-savings for the NHS.

Although it was inspired by local practice, we found the clinicians in the Falls project team did not think that the NHS ever really drove or owned the project. The aim of the project changed early on, after new research was published that made the original idea redundant. As it progressed, it became clear that the researchers and end users in the team had different views on whether the project should be doing new research on falls or implementing existing evidence, and also who should fund the latter type of work. Some members thought that the continuation of the project was driven more by its emblematic status in the PenCLAHRC bid and the desire of the program managers to have something useful to report to NIHR, than it was to meet the information needs of local end users.

Another mechanism was whether there was a “meeting of minds” (M2) between the researchers and end users involved in the projects. Members of the Stroke and TXA teams thought that each of these projects had clear goals, with all the organizations involved seeing the work as a priority. In contrast, this mechanism was weaker in the PFMT and Falls projects. Although there was no sense of any conflict of goals within the PFMT team, there was some internal debate between the clinicians in the project team and the senior managers in PenCLAHRC over the emergent design of the project. This was over whether to scale up or largely repeat the precursory PhD study it was based upon, and whether to publish the protocol for the study. In the Falls project, there was external support for the project at a strategic level in the partner organizations. However, members reported that within the team there was neither a “meeting of minds” over the purpose of the project nor a “common will” to find a mutually acceptable way forward.

A third mechanism was whether the members of the projects exhibited an active “knowledge appetite” (M3). This was apparent in the Stroke and TXA projects, where there was a sustained interest in learning through being part of the collaboration. For example, the ambulance trust sent some of its staff to a PenCLAHRC evidence-based practice course to improve its research capacity, and it subsequently became involved in the TXA and other projects. In the Stroke project, the clinicians had not undertaken operational research before, but they quickly saw the wider potential of the methodology. This led to further modeling of other aspects of the stroke clinical pathway in spin-off projects involving the same partners. Conversely, some of the clinicians in the Falls project intimated that their poor experience had diminished their appetite for similar work or left it unfulfilled.

The fourth mechanism linked to Rule 1 was one we called “game changers” (M4). This was where members of some projects found the experience of working together to be revelatory and transformative, facilitating new and more productive ways of doing and implementing research. For instance, researchers in the Stroke project were initially denied access to a data set they wanted to use for modeling purposes but gained access to another through the lead clinician, without whom they claimed this would have been impossible. They also found it easier than in the past to gain access to routine data they needed through the influence of the same clinician. The lead clinician in turn found it easier to “sell” the project to colleagues by using the researchers’ models, rather than “personal hunches,” to demonstrate the potential “real-life” benefits of making changes to the stroke pathway. Some of the clinicians also found that the process of helping to build the model served to shift their discussions from “the usual blame game” to the real scenarios they encountered and how they could be better managed.

The same mechanism was also active in the TXA project. Here managers in the ambulance trust had sufficient confidence in the strength of the evidence to write to all 11 emergency departments in the southwest, informing them of their intention to start administering TXA unless there were any objections. Previously, they would have waited for each department to agree individually, which would have taken longer. The trust was also encouraged by PenCLAHRC to publish its project documentation and tools online, to make it easier for other trusts to access, which again was not their usual practice.

### Rule 2: Establish Small Strategic Teams Led by Strong Facilitative Leaders

Over the 5 years of the pilot program, 148 projects were carried out under the auspices of PenCLAHRC. Each project team was usually comprised of a project lead, an executive lead (one of the senior managers of the program), a project facilitator, clinicians from the NHS trusts involved in the project, and one or more researchers with relevant methodological skills. Around half the projects also had some form of patient and public involvement. The teams met regularly and reported to the PenCLAHRC executive at intervals. Other people, including experts based at other universities in the United Kingdom, were sometimes intermittently involved in the projects.

In the case studies, we identified two related mechanisms underpinning our second simple rule concerning the leadership and configuration of the teams. One mechanism was the “facilitative leadership” (M5) style of the project leads. Members of the Stroke and TXA teams in particular thought that their leads had very good connections with colleagues within and outside the region. They were also commended for their vision, drive, enthusiasm, inclusiveness, and tenacity.

For example, in the Stroke project, leadership was shared between a physician and a researcher. The clinical lead developed the research question, facilitated access to a research data set and to routine data, was well connected with local and national research networks, and was actively involved in disseminating the work to other centers in the southwest. The research lead suggested operational research as the approach for addressing the question, contacted colleagues in other universities to draw on their methodological expertise in the analysis of some of the data, and supervised a researcher employed to carry out and help disseminate the research.

In the TXA project, it was the PenCLAHRC director’s chance meeting with an ex-colleague, who was the lead of a major international trial of TXA, which led to the project. The director brought together the team, acting on the new relationship formed with clinicians from the local ambulance trust through the capacity-building work of the program. The director also acted as the overall lead for the project, using his contacts with national experts and organizations to help address queries about the use of TXA in children. Meanwhile, the clinicians in the ambulance service and emergency departments led the actual implementation, developing the protocols for their colleagues to use on the ground. The clinical lead from the ambulance trust was also a member of a national group that subsequently helped to promote the implementation of TXA for trauma patients nationally in England.

In contrast, the leadership of the PFMT team was less well defined at the beginning. Eventually, a senior and respected clinician based in secondary care emerged as the lead. However, members thought that having an additional lead from primary care, who had inside knowledge of how to access funding for service redesign and who could champion the project from within primary care, might have been helpful. This was because the progress of their project had been badly affected by the national reorganization of primary care that occurred when they were seeking approval for the training package to be introduced and evaluated in a number of general practices. The changes had disrupted the application process and stalled the work for a few months. Similarly, in the Falls project, members described the leadership as changing over time. They also thought that the team lacked a clinical lead from primary care with the relevant credentials to lead a bid for external funding of a research project on the topic.

The other mechanism we found was whether the teams had a “small strategic core” (M6) of members. This was active in both the Stroke and TXA projects, which had what were described as small but complete teams. In contrast, the PFMT team was small and united but, as noted above, was not regarded as being complete. Membership of the Falls project team changed over time and relationships were strained. Some of the clinicians were unsure of their part in the team. The project meetings were also described as being large and long. After about 14 months, the size of the team was reduced when it was recognized that it had become too big.

### Rule 3: Harness and Develop Respective Assets

As described above, PenCLAHRC employed various strategies intended to increase local capacity for AHR. In the case studies, we identified a related mechanism about harnessing and developing assets in the NHS and academia that forms the basis of our third rule. Through a mechanism that we called “creative assets” (M7), participants were enabled by PenCLAHRC to harness existing and develop new assets for collaborative AHR. By “assets,” we mean a wide range of resources, such as people who have particular knowledge and skills; courses and workshops; good quality routine data that can be joined up and used for research and evaluation purposes; project management systems; and websites for sharing learning.

This mechanism was active in the Stroke and TXA projects. For example, the physician who proposed the Stroke project had been interested in the idea for over a year before PenCLAHRC was established. It was only after PenCLAHRC began, when the clinician was part-funded by the program and new researchers with expertise in modeling were appointed, that the project became viable. In the TXA project, the local ambulance trust built up and shared assets online in the form of a protocol, known as a Patient Group Direction, for paramedics to use to administer TXA in trauma cases.

Although none of the bids for funding associated with the Falls project were successful, some of the researchers (but not the clinicians) thought that the project had led to some valuable publications, including quantitative and qualitative systematic reviews of the literature on the topic. They also thought the project had increased local interest in and capacity for pursuing research on the wider topic of frailty among the elderly.

### Rule 4: Promote Relational Adaptive Capacity

An important part of the PenCLAHRC model was to involve local end users in the conduct and implementation of research and together to promote the use of that evidence elsewhere. To this end, the project leads and members used their connections within and outside the region to encourage the wider uptake of the research by their colleagues in other centers. We found this mechanism of “relational adaptive capacity” (M8) to be active in the Stroke and TXA projects.

As an operational research venture, the Stroke project involved building a model of part of the local stroke clinical pathway using clinicians’ knowledge of routine practice and good quality data. The model provided end users with an idea of the scale of benefits to be gained by making particular changes to the pathway. Any changes that were implemented on the basis of the model were then evaluated to examine the actual impacts. To spread this work, both the clinical lead for the project and the main researcher were actively involved in disseminating the results to other stroke centers in the region. They recognized that as each center had its own variation of the pathway, the modeling would need to be tailored to fit other localities. They also believed that this adaptive approach was more likely to be successful in centers where there was an equivalent clinical lead or operational manager prepared to champion the project in that setting.

In the TXA project, clinicians from the local ambulance trust employed a similar strategy. They developed a Patient Group Direction for the administration of TXA by paramedics in the region and then actively disseminated it (along with associated documents and a tool for costing the implementation) to other ambulance trusts nationally. They also recognized that the protocol might need to be adapted to suit local circumstances. The strategy was successful, as the Patient Group Direction was rapidly taken up nationally by most of the ambulance trusts in England. Two trusts in London were the last to do so because, it was suggested, of the closer access to hospitals where the drug could be administered within the recommended time frame. Similarly, the clinicians from the two local emergency departments developed a protocol for administering the drug in their centers and then promoted its adoption in other emergency departments across the region.

There were, however, notable differences in the clinicians’ experiences of implementing the Patient Group Direction in the ambulance service compared to the protocol in the local emergency departments. Paramedics were trained to follow a Patient Group Direction where indicated and this was an accepted part of their practice. Although clinicians also used protocols in emergency departments, we were told that, in complex trauma cases, they might have several protocols to follow and so have to use discretion in applying them, or might even forget to apply them (especially if, as in the administration of a drug such as TXA, the impacts on mortality and morbidity are not immediately apparent). For these reasons, the process of encouraging the clinicians in 11 emergency departments in the southwest to adopt and use the protocol was much more difficult. It took longer to achieve than it did for one large ambulance trust to implement the Patient Group Direction across its entire service.

In the PFMT and Falls projects, some members suggested that the interventions in these cases were particularly difficult to evaluate and translate to other places. This was because they were concerned with prevention (as well as treatment in PFMT); they also spanned multiple primary and secondary care organizations. For example, in the Falls project, the nature of the intervention in the original general practice that had a much lower rate of falls was seen by some to be hard to define because it involved many small and simple measures. Whether the same intervention could in principle be rolled out to other settings was also doubted because of variations in the local configuration of services and the populations served by general practices.

### Rule 5: Remember—The End User Is King!

Last, but not least, our final rule is based on a mechanism that we dubbed the “end user is king!” (M9). This mechanism relates to the team members’ attitudes to the involvement of end users in the projects and if (and when) they realized these were critical. We found it was most active in the Stroke and TXA projects.

In the Stroke project, there was a strong emphasis from the beginning on showing the clinicians whose support the project depended upon (including heads of departments and the clinicians on the ground who were not part of the project teams) the “real-life” issues that the research was concerned with and the potential gains to be made. As one clinician in the team put it, it was important that the proposed research “meant something” to them. With this in mind, the team involved a wide range of clinicians from the hospital stroke unit and the ambulance trust in building the model, and they carried out additional modeling of the stroke pathway to help persuade them of the relevance of the research.

However, at an interim presentation of the work to clinicians in the emergency department, who had not been as involved in developing the research question or in the modeling to date, the modelers encountered some resistance and queries about the construction of the model. Although they dealt with the issues, the modelers realized that they should have engaged with more of these clinicians earlier in the project. They described how they had learned from this experience and planned to engage a relevant mix of clinicians earlier in future operational research projects.

In the TXA project, although the published evidence for using TXA in trauma was strong, it had not been written from an ambulance service perspective. It was converted into a Patient Group Direction that paramedics could routinely follow by the implementation lead from the ambulance trust. A draft of this document was also circulated to the trust’s established consultation group for comments before the final version was instituted locally. The protocol and associated documents were then published online to make it easier for other ambulance trusts in England to access and adapt them to their own regions, which they did quite quickly.

Relations with end users were less strong in the other projects. Although there was support for the PFMT project from the clinical commissioning group, it was still depicted as being fragile by members because of its dependence on, and PenCLAHRC’s weak links with, the primary care organizations involved. The Falls project had the support of the most senior managers in the partner NHS trusts but it was clear that some of the clinicians did not see the project as fitting their needs and agendas.

## Discussion

In this article, we have described the results of an evaluation of PenCLAHRC’s “interactive” approach to promoting knowledge translation. We focused on the program architects’ theory that research driven by end users, and carried out by researchers in partnership with them, would produce evidence that was more likely to meet end users’ information needs than traditional ways of doing research. This theory was tested in four case studies of projects carried out by PenCLAHRC, in which we examined how closer collaboration was enacted and whether any relevant and useful knowledge was produced and implemented by the end users to date.

We found that the Stroke and TXA projects did quickly achieve their knowledge translation goals whereas the PFMT and Falls projects struggled, despite the considerable efforts of the teams concerned. When we examined how closer collaboration was enacted in these projects, we identified 9 mechanisms that seemed to make a difference to their success. Whether or not the mechanisms were active was contingent on various contextual factors that modulated the participants’ efforts to achieve what they set out to do.

Drawing on complexity theory, we summarized these mechanisms in the form of 5 simple rules that characterized successful closer collaboration in PenCLAHRC. In our “black box” model, it is possible that any one of the 9 mechanisms could, if activated, initiate change. However, we think that together they are more likely to create a momentum for change and overcome the inertia of the systems that PenCLAHRC was contending with. For example, it is arguable that the participants in the Stroke and TXA projects were enabled, through the range and strength of the mechanisms they activated, to more effectively anticipate issues and take preemptive or remedial action, seize moments, and address problems as they arose. This was in contrast to the PFMT and Falls projects, where participants were more susceptible to or entrenched in the state of the systems that they had less leverage over.

Based on our observations of how researchers and end users collaborated on the projects, we have modified the working proposition we started with as follows:

Research and implementation projects that are coproduced by researchers and professional end users are more likely to generate new evidence or adapt existing evidence in ways that meet these end users’ specific information needs. Successful coproduction also whets the same partners’ wider appetite for further collaboration on new projects. Together they may also help to promote the adaptation of the evidence in kindred settings by other potential end users with similar information needs.

Our use of the term “coproduction” in this proposition was also informed by our wider knowledge of the literature on this concept. Although the program architects and participants did not use the term themselves, we noted that the mechanisms of closer collaboration that we observed were largely consistent with the principles of coproduction. Below we discuss the relevance of this existing social theory and the insights it provides into why the ways in which this style of closer collaboration might facilitate knowledge translation.

According to Ostrom (1996), coproduction is “a process through which inputs used to produce a good or service are contributed by individuals who are not ‘in’ the same organization” (p. 1073). Generally, the term has been used to describe a type of relations between the providers of goods or services (such as the state) and consumers or users of them (the public). Here we suggest that it can also be used as a mid-range theory to describe similar relations between the producers of knowledge (traditionally academics) in the form of AHR and end users of that knowledge (who include not only patients and the public but also policy makers, health service commissioners, managers, and clinicians).

Relations of coproduction are characterized by a number of elements or principles (Boyle & Harris, 2009; Social Care Institute for Excellence, 2013). Five of the elements commonly used to define it are listed in Table 3, along with our description of how they apply to relations of knowledge production and utilization. As it shows, these elements are all broadly compatible with the mechanisms and associated simple rules we described above. The one exception is the “small strategic core” (M6) mechanism concerning the size of the team, which is a topic that is less well defined in the literature on coproduction.

**Table 3.** Basic Elements of Coproduction, Applied to Producers and End Users of Knowledge.

Elements	Description
Active agents	End users of knowledge are active contributors to and cocreators of knowledge, not passive recipients of research conducted by others for them
Equality of partners	There is a shift in the balance of power, with research becoming more end user driven. Researchers and end users have equally valued contributions to make to the conduct and application of research
Reciprocity and mutuality	End users and researchers can each provide something that the other needs; each benefits from the relations. The partners are committed to each other
Transformative	End users’ and researchers’ respective needs and goals are met; they make more and better use of resources; they develop capacity and social capital; the distinction between researchers who produce knowledge and end users who apply it is blurred
Facilitated	Relevant networks and infrastructure incentivize and support coproduction relations, and develop and mobilize knowledge and capabilities

What is particularly relevant about the theory of coproduction is that it is founded on the notion that the success of social programs is critically influenced, one way or the other, by the acceptability of the program to end users. In PenCLAHRC, the leads in both the Stroke and the TXA projects were particularly attuned to this. As we showed, they paid particular attention to the “real-life” concerns and needs of the clinicians who were not part of the project teams but who were the agents on the ground critical to the implementation of stroke thrombolysis and TXA in trauma (i.e., the clinicians in the hospital’s stroke and emergency departments, and the paramedics). They also made considerable efforts to engage and involve these clinicians and their managers at all levels. Through the support of the program, the teams were also able to promote the findings to other receptive end users and help to facilitate the implementation of the evidence more widely.

These findings add to previous understanding of partnership programs by showing how some projects in PenCLAHRC achieved knowledge translation through a style of closer collaboration that was largely congruent with the principles of coproduction. This approach to knowledge translation is consistent with a move away from simple models based on unidirectional concepts of “knowledge transfer” and bidirectional concepts of “knowledge exchange” toward a more dynamic view of the uncertain, complex, and contextually contingent ways in which knowledge is created and applied by researchers and end users (Davies, Nutley, & Walter, 2008; Mitchell et al., 2009). Crucially, this approach recognizes the different insights, experience, and skills that researchers and end users can contribute to projects, as well as the importance of the meaning and acceptability of the work to the wider end users on the ground.

### Strengths and Limitations

One of the strengths of this evaluation was that it too was coproduced with the participants in PenCLAHRC. Through its participatory design, senior managers were actively involved in helping to specify the program theories that were examined in depth by the evaluation team, and in the selection of the projects that served as cases for testing these theories. As members of an internal formative evaluation team, we were able to periodically provide feedback information to the senior managers and help shape the emergence of the program. Although the senior managers of the program were involved in the evaluation, we worked autonomously and arrived at our conclusions independently and without any interference. Our position as internal evaluators enabled us to observe the program unfold daily over the lifetime of the program, and to carry out a detailed and credible study of the theories underpinning the program, from an insider perspective.

By adopting a theory-driven approach, we were able to elicit and test the program architects’ and participants’ folk theories about how the program and projects were supposed to work, as well as to draw on formal social theory on coproduction to help explicate the results. The qualitative methods of data collection and analysis that we used enabled us to describe in depth the contexts in which the program and projects operated, as well as the ways in which closer collaboration was enacted by the teams. Through our synthesis of the findings of the case studies, we were able to specify the key mechanisms of closer collaboration that were critical to the success of the projects and to summarize them in the form of five simple rules. This information should help others to assess the relevance and potential transferability of the findings of this evaluation of the “interactive” model developed by PenCLAHRC to other settings.

A limitation of the evaluation was that we only examined the coproduction of research with professional end users, and not patients and the public, who were also partners in PenCLAHRC and who were involved in around half the projects by the end of the pilot program. When we selected the case studies, with the help of the senior managers of the program, we purposefully did not specify patient and public involvement in our criteria as there were other embedded evaluations examining this in PenCLAHRC, and we did not want to burden these participants (although we did not exclude them from consideration).

We also focused on the theories of closer collaboration at the possible expense of other theories and allied mechanisms also at work in the “black box” that were beyond the scope of this study. As Greenhalgh et al. (2009) have noted, realistic evaluation does involve the difficult task of deciding which of the many candidate theories to select for empirical study. After discussing different options, we decided to focus in depth on closer collaboration rather than carry out a broader study of a wider range of theories and associated mechanisms. We also limited the evaluation to the four case studies and did not have the resources or time to examine more, nor to follow up the emerging spin-off projects and role of the members of the Stroke and TXA projects in facilitating the wider uptake of the evidence by clinicians in other localities.

We also acknowledge that, at the time of writing, the outcomes of three of the four projects are still unfolding. Over the 5 years of the evaluation, it was possible to observe the changes to the design of NHS services made as a result of the Stroke and TXA projects, and the frustrated efforts of the Falls team to reconfigure the project. However, the PFMT project was seriously delayed by national changes in the organization of primary care and had not properly got underway by the end of the evaluation. The longer term impact of the service changes from the Stroke, TXA, and PFMT projects on patients’ health outcomes is still to be observed. It can take several months for a sufficient number of patients to have been recorded using the redesigned services for statistical analysis to be completed and the results made available for program evaluations such as this.

Notwithstanding the above limitations, we hope that this article has provided some useful insights into the mechanisms of closer collaboration that we discerned within the “black box” of PenCLAHRC and the simple rules of coproduction that underpinned them. These rules provide a possible heuristic for future architects and executors of collaborative programs such as the CLAHRCs and those involved in the production and utilization of AHR more generally.

Further research is required to examine if and how involving patients and the public, as well as professionals, in the coproduction of research and implementation projects is advantageous in closing the second translational gap in research and clinical practice. This study has also highlighted a case for developing and evaluating strategies for coproduction in translation, where evidence coproduced by partners in one setting is actively shared with, taken up, and adapted by receptive colleagues in another. It would be useful to examine how this process might be expedited by infrastructures such as the CLAHRCs and AHSNs supporting a distributed model of coproduction of AHR, bringing together constellations of original and new groups of collaborators to further spread and embed innovation.
